# The Ubiquitin Proteasome System and Nutrient Stress Response

**DOI:** 10.3389/fpls.2022.867419

**Published:** 2022-05-19

**Authors:** Erin Mackinnon, Sophia L. Stone

**Affiliations:** Department of Biology, Dalhousie University, Halifax, NS, Canada

**Keywords:** ubiquitination, 26S proteasome, ubiquitin ligase, protein degradation, abiotic stress, nutrient stress, nutrient uptake

## Abstract

Plants utilize different molecular mechanisms, including the Ubiquitin Proteasome System (UPS) that facilitates changes to the proteome, to mitigate the impact of abiotic stresses on growth and development. The UPS encompasses the ubiquitination of selected substrates followed by the proteasomal degradation of the modified proteins. Ubiquitin ligases, or E3s, are central to the UPS as they govern specificity and facilitate the attachment of one or more ubiquitin molecules to the substrate protein. From recent studies, the UPS has emerged as an important regulator of the uptake and translocation of essential macronutrients and micronutrients. In this review, we discuss select E3s that are involved in regulating nutrient uptake and responses to stress conditions, including limited or excess levels of nitrogen, phosphorus, iron, and copper.

## Impact of Environmental Factors on Nutrient Acquisition

Plants must cope with external factors that impact the uptake of nutrients, which are essential for growth, development, and yield. Climate change poses additional challenges to the availability and acquisition of nutrients, further impacting plant health. According to the Intergovernmental Panel on Climate Change (IPCC), global temperature is projected to increase 1.0°C (highest mitigation efforts) to 5.7°C (lowest mitigation efforts) by the end of this century (IPCC, 2021). With climate change, extreme weather events occur more frequently, and the duration and timing of these events become more erratic. High temperatures contribute to drought conditions in many habitats (Mimura, 2013). Increased water vapor in the atmosphere from high temperatures contributes to flooding (Sun et al., 2007; O’Gorman and Schneider, 2009). Rapid warming of the arctic and atmospheric pressure changes cause disruptions to the polar vortex, leading to irregular temperature/weather patterns and colder climates in ecosystems that are not acclimated to low temperatures (Cohen et al., 2020; Overland, 2021). In addition to directly affecting plant health, these climatic shifts affect growth *via* the alteration of soil properties such as pH, which, among other issues, impacts the acquisition of nutrients.

Soil temperature and moisture are two important determinants in the availability and uptake of nutrients. Temperature extremes impact nutrient acquisition by influencing root growth, soil microbial diversity, diffusion of nutrients across the soil, and the level of nutrients available for uptake (Baon et al., 1994; Pregitzer and King, 2005; Yan et al., 2013; Gelybó et al., 2018; Maharajan et al., 2021). For example, low temperatures reduce phosphorus (P) uptake in corn (*Zea mays*; Mackay and Barber, 1984; McCallister et al., 1997; Maharajan et al., 2021). Similarly, elevated temperature has been shown to decrease P uptake in wheat (*Triticum aestivum*; Kumar et al., 2012; Maharajan et al., 2021). Elevated temperatures and heavy rain caused by climate change has made P deficiency one of the leading restrictive factors for crop growth (López-Arredondo et al., 2014). Root colonization by arbuscular mycorrhizal fungi such as *Glomus mosseae* has been shown to promote uptake of nutrients including P in barley (*Hordeum vulgare*) and zinc (Zn) in red clover (*Trifolium pratense*; Baon et al., 1994; Chen et al., 2003; Wu and Zou, 2010). Low temperate has been shown to reduce mycorrhizal formation limiting the beneficial effects of the fungus on nutrient uptake (Baon et al., 1994; Wu and Zou, 2010). High temperatures contribute to dry soil conditions, which reduces the rate of nutrient diffusion from the rhizosphere to the absorbing surface of the roots (Pregitzer and King, 2005).

Soil salinity is one of the major abiotic factors limiting crop production. Plants grown under high salinity conditions display reduced content and uptake of essential nutrients including P, Zn, nitrogen (N), potassium (K), calcium (Ca), and iron (Fe; Brown et al., 2006; Bano and Fatima, 2009; Etesami and Noori, 2019). Water logging increases soil leaching resulting in loss of nutrient cations and salts, as well as higher soil acidification (Karmakar et al., 2016; Gelybó et al., 2018). Erosion caused by heavy rainfall also depletes soil nutrients (Yao et al., 2021). Plants have an optimal soil pH range for maximum growth, and pH above or below this range has been shown to influence nutrient uptake and content (Islam et al., 1980; Haynes and Swift, 1986; Neina, 2019). Blueberry (*Vaccinium* spp.) grown in soil with pH above optimal (4.0–5.5) had reduced micronutrient (copper [Cu], manganese [Mn], Zn, and Fe), and macronutrient (magnesium [Mg], K, P, Ca) content in leaves (Jiang et al., 2017). Soil pH is also a major determinant of the level of nutrients available for use by plants. For example, Zn and Cu are more readily available in acidic soils (Kabata-Pendias, 2004). Nutritional status is not only essential to plant health but is also important for coping with adverse environments as the detrimental effects of abiotic stresses may be minimized by optimizing nutrition, which influences water circulation, photosynthesis, and other physiological processes (Ahanger and Ahmad, 2019).

Climate change, in addition to the increasing global population, puts immense pressure on agricultural productivity, increasing the urgency for understanding the molecular basis for plant response to abiotic stresses. Plants rely heavily on regulatory mechanisms such as the ubiquitin proteasome system (UPS) to maintain cellular homeostasis and continued growth under adverse conditions. The UPS is used to regulate the function of proteins involved in generating the cellular changes required to respond to the changing environment and mitigate the negative impact of stress (Miricescu et al., 2018; Stone, 2019). This review will discuss the role of the UPS in facilitating nutrient uptake, as well as various components of the UPS which have known or predicted roles in responding to nutrient stress.

## The Ubiquitin Proteasome System

**Figure 1:**
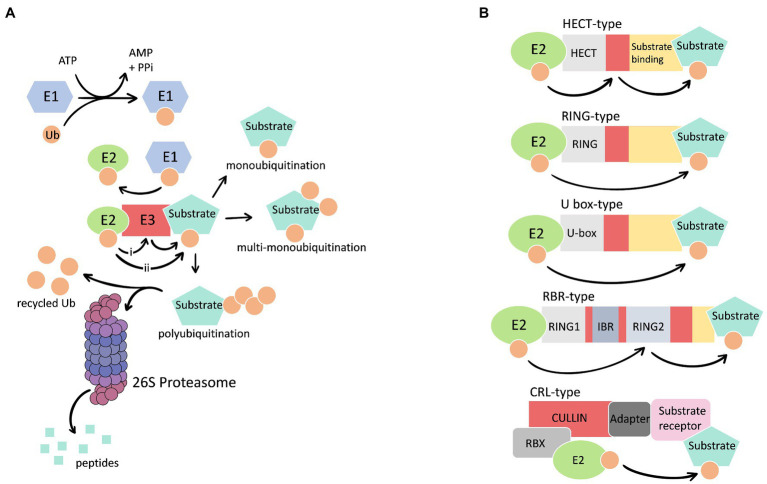
**(A)** A simplified outline of the ubiquitin proteasome system. The E1, E2, and E3 enzymes facilitate attachment of one or more ubiquitin (Ub) molecules to the target substate. Ubiquitination of the target occurs through transfer of Ub from the E2 to the E3 active cysteine prior to attachment to the substrate (i) or direct transfer of Ub to the substrate (ii). The conjugation cascade results in the monoubiquitination (one Ub at one site), multi-monoubiquitination (multiple Ubs at different sites), or polyubiquitination (multiple Ubs forming a chain) of the substrate. Polyubiquitinated substrates are recognized and degraded by the 26S proteasome. Ub is removed from the substrate and recycled. **(B)** Schematics representation of different E3 types. E3s utilize a RING (Really Interesting New gene), HECT (Homologous to E6AP C-terminus), or U-box domain to interact with the E2. Single subunit HECT and RING-in-between-RING (RBR) type E3s accept the Ub from the E2. Complex E3 Cullin (Cul)-RING ubiquitin ligases (CRLs) utilize different subunits to interact with the E2 and substrate.

The UPS involves the ubiquitination of a selected substrate followed by proteasomal degradation of the modified protein (Figure 1A). Ubiquitination is the covalent attachment of ubiquitin (Ub), a small, highly conserved protein, to substrates. The conjugation process requires the sequential actions of three types of enzymes: ubiquitin activating enzyme (E1), ubiquitin conjugating enzyme (E2), and ubiquitin ligase (E3). Degradation of the ubiquitinated protein is accomplished by the 26S proteasome, a large multi-catalytic multi-subunit complex. The system allows plants to efficiently regulate almost every aspect of cellular function *via* the degradation of numerous proteins including enzymes, transporters, ion channels, signaling proteins (e.g., kinases and receptors), and transcription regulators (e.g., transcription factors, co-activators, and repressors; Trujillo and Shirasu, 2010; Sadanandom et al., 2012; Adams and Spoel, 2018; Stone, 2019). In response to external stimuli, ubiquitination can be promoted or inhibited leading to increased degradation or stabilization of a substrate protein, respectively. These changes in protein abundance may facilitate or prohibit cellular responses.

The E1 initiates the enzymatic cascade, creating a thioester bond between the active site cysteine and the C-terminal glycine residue of Ub in an ATP-dependent reaction (Figure 1A). Ubiquitin is then transferred from the E1 to the active site cysteine of the E2, forming a thioester linked E2-Ub intermediate. The E3 mediates transfer of Ub from the E2 to the substrate, *via* the formation of an isopeptide bond between the Ub C-terminal carboxyl group and the amino group of a residue on the substrate, typically a lysine side chain. Substrate specificity is attributed to a large and diverse collection of E3s. For example, the *Arabidopsis thaliana* (Arabidopsis) genome is estimated to encode for over 1,500 single subunit E3s or components of E3 complexes (Vierstra, 2012). Single subunit ubiquitin ligases include enzymes that utilize a Really Interesting New Gene (RING), Homologous to E6AP C-Terminus (HECT), or U-box domain to interact with the E2-Ub intermediate (Figure 1B). Complex E3s such as the Cullin (Cul)-RING ubiquitin ligases (CRLs) consists of a scaffold Cul protein interacting with a substrate-recognition component, with or without an adaptor subunit, and an E2-binding RING-Box (Rbx) protein (Figure 1B). Except for the single subunit HECT-type and RING-in-between-RING (RBR)-type E3s that accept Ub from the E2, ubiquitin ligases facilitate the transfer of Ub from the E2 directly to the substrate (Figure 1B). The pervasiveness of ubiquitin-dependent regulation is due in part to a single E3 regulating the abundance of multiple substrates. Additionally, depending on the environment, multiple E3s may target a particular substrate for ubiquitination.

The conjugation cascade can result in monoubiquitination (attachment of one Ub), multi-monoubiquitination (attachment of a single Ub to two or more sites), or polyubiquitination (attachment of a polyubiquitin chain) of the substrate (Figure 1A). There is a degree of structural plasticity in polyubiquitination due to flexibility of chain conformations caused by the different linkages used to generate Ub chains. Ub-Ub linkages can be created using eight different attachment sites, including seven lysine residues (6, 11, 27, 29, 33, 48, and 63) and the N-terminal methionine of ubiquitin (Dittmar and Winklhofer, 2020). The different chain configurations influence the fate of the modified substrate. For example, a chain created using 63-lysine is linked to non-proteasomal outcomes such as endocytosis, while a chain generated using 48-lysine serves as a signal for degradation by the 26S proteasome (Thrower et al., 2000; Varadan et al., 2002; Duncan et al., 2006). The attachment of Ub is reversible *via* the actions of deubiquitylating enzymes (DUBs) that act as alternate regulators of ubiquitination, which cleave the isopeptide bond between Ub molecules to shorten chains and remove ubiquitin from substrate proteins (Komander, 2010). The best-known function of ubiquitination is targeting proteins to the 26S proteasome for degradation (Figure 1A).

The 26S proteasome is a compartmentalized complex composed of a hollow cylindrical 20S Core Particle (CP), which is capped at one or both ends by a 19S Regulatory Particle (RP; Figure 1A; Bard et al., 2018). The CP consists of four stacked heptameric rings with protease activities found on the β subunits of the two inner rings. The RP recognizes and removes the ubiquitin/ubiquitin chain from the substrate, unfolds, and directs the protein into the chamber of the CP for proteolysis. The removed ubiquitin molecules are recycled and the 26S proteasome expels the resulting peptides.

## The UPS and Nutrient Stress

The UPS is an important regulator of plant responses to abiotic stresses such as drought, heat, and salinity (Stone, 2019; Melo et al., 2021). The expression of genes that encode for components of the UPS are induced by abiotic stresses and mutations that hinder activity of ubiquitin enzymes or the proteasome have been shown to alter stress tolerance (Wang et al., 2009, 2019; Sun et al., 2020). Ubiquitin-dependent proteolysis is an important regulator of the acquisition of nutrients from the rhizosphere. Failure to properly regulate nutrient uptake is detrimental to growth and development, increases susceptibility to diseases, and reduces abiotic stress tolerance (Huber et al., 2012; Ahanger and Ahmad, 2019). The UPS is intricately involved in regulating nutrient acquisition *via* modulating the abundance of transcriptional regulators that control the expression of nutrient-responsive genes, components of nutrient signaling pathways, specialized channels, and transporters that uptake and translocate nutrients (Table 1). Factors such as nutrient deficiency may increase the ubiquitin-dependent degradation of a transcriptional repressor to promote the expression of nutrient stress-responsive genes or decrease the turnover of a membrane-bound transporter to enhance uptake. Alternatively, the UPS may promote the degradation of transcriptional activators and transporter proteins to attenuate the uptake of nutrients. Here, we describe some of the available evidence to illustrate the essential regulatory role of the UPS in the acquisition of macronutrients: N, P, and K; and micronutrients: Fe, Cu, and boron (B; Table 1).

**Table 1:** Ubiquitin enzymes involved in nutrient uptake and stress response.

Ubiquitin enzymes		Function	Substrate (Known or potential)	Species	References
ATL8 (Arabidopsis Tóxicos en Levadura 8)	RING E3	Phosphate deficiency response	Unknown	*Arabidopsis thaliana*	Ramaiah et al., 2022
ATL31 (Arabidopsis Tóxicos en Levadura 31)	RING E3	Carbon/Nitrogen balance	14–3-3χ	*A. thaliana*	Sato et al., 2011
BTS (BRUTUS)	RING E3	Iron deficiency response	PYEL (Popeye-like)	*A. thaliana*	Selote et al., 2015
BTSL1/BTSL2 (BRUTUS-like 1/2)	RING E3	Iron deficiency response	FIT (FER-like iron deficiency-induced transcription factor)	*A. thaliana*	Rodríguez-Celma et al., 2019
CPN1 (Copine 1)	RING E3	Na^+^/K^+^ Homeostasis	SKD1 (Suppressor of K^+^ transport Growth Defect 1)	*Mesembryanthemum crystallinum*	Chiang et al., 2013
GmARI1 (*Glycine max* ARIADNE 1)	RBR E3	Aluminum toxicity	Unknown	*G. max*	Zhang et al., 2014
HRZ1/HRZ2 (Hemerythrin motif-containing RING- and Zinc-finger protein 1/2)	RING E3	Iron stress impacts zinc uptake	PRI1 (Positive regulator of iron homeostasis 1)	*O.sativa*	Kobayashi et al., 2013
IDF1 (IRT1 degradation factor 1)	RING E3	Iron deficiency response Impacts uptake of other cations (zinc, cobalt, manganese, cadmium)	IRT1 (Iron-regulated transporter 1)	*A. thaliana*	Shin et al., 2013
NBIP1 (NRT1.1B interacting protein 1)	RING E3	Nitrogen stress	SPX4	*Oryza sativa*	Hu et al., 2019
NLA (Nitrogen limitation adaptation)	RING E3	Nitrate acquisition	NRT1.7 (Arabidopsis nitrate transporter 1.7)	*A. thaliana*	Liu et al., 2017
		Phosphate acquisition	PHT1;4 (Phosphate transporter 1;4)	*A. thaliana*	Lin et al., 2013; Park et al., 2014
		Nitrogen deficiency response	ORE1	*A. thaliana*	Park et al., 2018
PIE1 (Pi starvation-induced E3 ligase)	RING E3	Phosphate deficiency response	SPX2	*O. sativa*	Yang et al., 2018
PHO2 (Phosphate 2)	UBC/E2	Phosphate deficiency response		*A. thaliana*	Lin et al., 2013;Park et al., 2014
PRU1 (Phosphate response ubiquitin E3 ligase 1)	F-Box CRL E3	Pi-deficiency response	WRKY6	*A. thaliana*	Ye et al., 2018
RGLG1/2 (RING Domain ligase1/2)	RING E3	Iron deficiency response	Unknown	*A. thaliana*	Pan et al., 2015
SDEL1/2(SPX4 degradation E3 ligases 1/2)	RING E3	Phosphate deficiency response	SPX4	*O. sativa*	Ruan et al., 2019
Unknown		Boron stress	BOR1 (Boron transporter 1)	*A. thaliana, O. sativa*	Yoshinari et al., 2021
Unknown		Iron deficiency response	AHA2 (H + -ATPase2)	*A. thaliana*	Martín-Barranco et al., 2020
Unknown		Iron deficiency response	FRO2 (Ferric chelate reductase 2)	*A. thaliana*	Martín-Barranco et al., 2020

### Macronutrients

Primary macronutrients (e.g., N and P) are required in large amounts relative to secondary macronutrients (e.g., Ca and Mg) and essential micronutrients.

**Figure 2:**
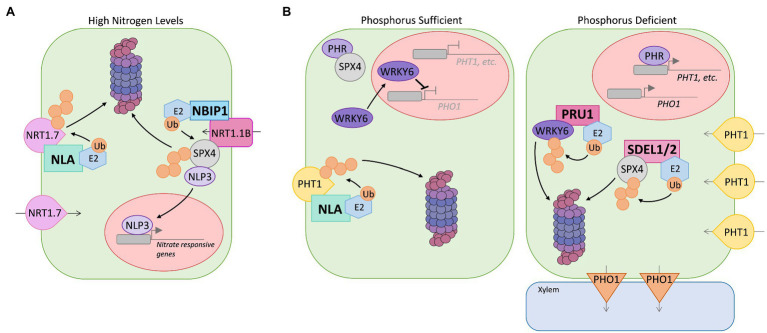
Simplified representation of the role of select E3s from Arabidopsis and *Oryza Sativa* (rice) in regulating nutrient uptake. **(A)** Under high N, the E3 NLA mediate ubiquitin-dependent degradation of NRT1.7 nitrate transporter to avoid N overaccumulation. The transreceptor NRT1.1B recruits the E3 NBIP1, which ubiquitinates SPX4 allowing the transcription factor NLP3 to enter the nucleus and promote expression of N-responsive genes. **(B)** Under Pi replete conditions, E3 NLA ubiquitinates PHT1 inorganic phosphate transporters facilitating degradation by the 26S proteasome to reduce uptake and prevent Pi overaccumulation. Under P limiting stress conditions, E3s SDEL1 and SDEL2 mediate the degradation of SPX4, which allows the transcription factor PHR1/2 to activate the expression of *PSI* genes such as *PHT1*. Also, the E3 PRU1 mediates degradation of the repressor WRKY6, which relives inhibition of *PHO1* transcription. Increase in PHO1 transporter abundance promotes loading of Pi into the root xylem.

*Nitrogen*: N is a major component of amino acids, nucleotides, and chlorophyll. Low N availability limits growth, development, and yield. Plants uptake inorganic forms of N as nitrate (NO_3_^−^) and ammonium (H_4_N^+^). Soil amino acids serve as an organic N form (Zhang et al., 2020). Arabidopsis has four families of nitrate transporters including the NRT1 family, which are predominantly low-affinity transporters involved in the sensing, uptake, and translocation of NO_3_^−^ (Tsay et al., 2007). The RING-type E3 Nitrogen Limitation Adaptation (NLA) is as a major component in the molecular machinery that regulates N deficiency response (Peng et al., 2007; Kant et al., 2011; Liu et al., 2017; Figure 2A). Under N deficiency, *nla* mutants display premature senescence indicating hypersensitivity to the stress (Peng et al., 2007). NLA, which is predominantly localized to the plasma membrane, mediates the ubiquitin-dependent degradation of NRT1.7 (Liu et al., 2017; Hannam et al., 2018; Figure 2A). The phloem expressed NRT1.7 is involved in source-to-sink remobilization of nitrate (Fan et al., 2009). *nrt1.7* mutants exhibited abnormally high levels of NO_3_^−^ in senescent leaves, suggesting NRT1.7 is an important facilitator of phloem loading to remobilize nitrate. NLA levels decrease during exposure to N limiting conditions, allowing for the increase in NRT1.7 abundance, which promotes N mobilization (Liu et al., 2017). In rice (*Oryza sativa*), the RING-type E3 NRT1.1B interacting protein 1 (OsNBIP1) targets the repressor protein SPX4 (named after *Saccharomyces cerevisiae* SYG1 and PHO81 and mammalian XPR1 [SPX] domain-containing proteins) for proteasomal degradation, which alleviates inhibition of transcription factor NLP3 to promote expression of N-responsive genes (Figure 2A) (Hu et al., 2019). The perception of nitrate by the transreceptor OsNRT1.1B is suggested to recruit OsNBIP1, which then ubiquitinates SPX4 (Figure 2A). The RING-type E3s, Arabidopsis tóxicos en levadura (ATL) 6 and ATL31, were identified as negative regulators of the response to changes in the balance of available carbon (C) to N during seedling growth (Sato et al., 2009 and 2011). The strict coordination of C to N levels (termed the C/N response) is critical to seedling success, inhibiting post-germinative growth under high C/low N stress conditions (Sato et al., 2009). The C/N response is also vital to ecosystem success, as an ideal C/N ratio in plants is necessary to optimize CO_2_ utilization (Zheng, 2009). The 14–3–3 protein, 14–3-3χ, accumulates in response to C/N stress and promotes early seedling growth arrest (Sato et al., 2011). Under high C/low N conditions, phosphorylation stabilizes ATL31, which interacts with and mediates the proteasome-dependent degradation of 14–3-3χ to attenuate the nutrient stress response (Yasuda et al., 2014 and 2017). Calcineurin B-Like (CBL)-Interacting Protein Kinase 14 (CIPK14), which is activated in a Ca^2+^-dependent manner *via* its association with CBL8, mediates phosphorylation of ATL31 under high C/low N stress, suggesting the involvement of Ca^2+^ signaling in the C/N response (Yasuda, 2017).

*Phosphorus*: P is a key component of macromolecules, and it plays a fundamental role in photosynthesis, metabolism of nitrogen, carbohydrates, and fat (López-Arredondo et al., 2014). Regulating the abundance of phosphate transporters is important as low P levels significantly reduce root growth and net photosynthesis, and high levels of the transporters cause toxic P accumulation in cells. For Arabidopsis, four families of Phosphate Transporters (PHTs), PHT1-PHT4, are involved in the intracellular transport and uptake of inorganic phosphate (Pi) from soil (Młodzińska and Zboińska, 2016). Of the nine PHT1 members, plasma membrane-localized PHT1;1, PHT1;2, PHT1;3 and PHT1;4 are primarily involved in the acquisition of Pi (Shin et al., 2004; Ayadi et al., 2015). A family of Phosphate Starvation Response (PHR) transcription factors regulate the expression of *Pi-starvation-induced* (*PSI*) genes including *PHT1s* (Bustos et al., 2010). In rice, OsSPX4 interacts with OsPHR2 under Pi-replete conditions, inhibiting transcriptional activity (Lv et al., 2014; Figure 2B). The RING-type ubiquitin ligases, SPX4 degradation E3 ligases 1 (SDEL1) and SDEL2, accumulate under Pi-deficiency and mediate the ubiquitin-dependent proteasomal degradation of the repressor, which would allow OsPHR2 to enter the nucleus and promote expression of *PSI* genes (Ruan et al., 2019; Figure 2B). Nitrate-induced degradation of SPX4, mediated by the E3 OsNBIP1, also contributes to the activation of OsPHR2 and expression of *PSI* genes (Hu et al., 2019).

The E3 NLA is an important regulator of phosphate homeostasis, modulating the abundance of PHT1s at the plasma membrane (Kant et al., 2011; Lin et al., 2013). Under phosphate sufficient conditions, loss of *NLA* function results in accumulation of PHT1s and increased Pi content causing toxicity. NLA, along with the E2 Phosphate 2 (PHO2 [or UBC24]), has been shown to mediate the ubiquitin-dependent proteasomal degradation of PHT1;4 to suppress Pi uptake under high phosphate conditions (Liu et al., 2012; Lin et al., 2013; Park et al., 2014; Figure 2B). NLA and PHO2 levels are kept low *via* the action of Pi starvation-induced microRNAs, allowing for the accumulation of phosphate transporters and increase Pi uptake (Bari et al., 2006; Kant et al., 2011; Lin et al., 2013). The increase in microRNAs under Pi-deficient conditions requires the transcription factor PHR1 (Bari et al., 2006). P homeostasis is also controlled *via* the activity of Phosphate Response Ubiquitin E3 Ligase 1 (PRU1), which promotes the ubiquitin-dependent degradation of WRKY6, a transcription factor known to inhibit transcription of *Phosphate 1 (PHO1*; Chen et al., 2009; Lin et al., 2013; Ye et al., 2018; Figure 2B). PHO1 is expressed under P deficiency and is involved in the translocation of phosphorus from root-to-shoot (Hamburger et al., 2002). PRU1 is a F-box protein, which function as the substrate-binding component for CRL E3 complexes (Ye et al., 2018). PRU1 promotes the proteasomal degradation of WRKY6 under low P stress to increase the abundance of PHO1, promoting the movement of the nutrient into root xylem (Figure 2B).

*Potassium*: K is an activator of many enzymes involved in protein synthesis, sugar transport, and metabolism of N and C (Tränkner et al., 2018). K also plays a key role in gas exchange and transpiration as optimal K levels are essential for stomatal opening and closing (Andrés et al., 2014). Maintaining K homeostasis is critical, as excess K negatively affects the uptake of other nutrients including N, Ca, and Mg (Tränkner et al., 2018). Plants uptake potassium ions (K^+^) *via* Shaker-like K^+^ channels, such as AKT1, and K^+^ transporters including AtHAK5 (Gierth et al., 2005; Rubio et al., 2008; Pyo et al., 2010). Under K^+^replete conditions, uptake is dominated by ATK1 and other low-affinity K^+^ uptake mechanisms (Rubio et al., 2010). K^+^ deficiency induces the expression of AtHAK5, a high-affinity transporter essential for uptake under limiting conditions (Ahn et al., 2004; Gierth et al., 2005; Pyo et al., 2010). The extent to which ubiquitin-mediated processes are involved mediating K uptake is unclear. Salt stress induces the expression of Suppressor of K^+^ transport Growth Defect 1 (SKD1), an AAA-type ATPase, which facilitates K^+^ transport to maintain Na^+^/K^+^ homeostasis (Ho et al., 2010). Studies using the halophyte ice plant *Mesembryanthemum crystallinum* found that the SKD1 is ubiquitinated by the RING-type E3 Copine 1 (CPN1; Chiang et al., 2013). Under high salt stress, SKD1 relocates from the cytosol to the plasma membrane where it interacts with CPN1 (Chiang et al., 2013; Jou et al., 2013).

### Micronutrients

Plants require very small quantities of micronutrients; but extremely low levels will cause deficiencies, and excess nutrient levels are toxic. Therefore, maintaining optimal levels of these nutrients are critical for plant success.

*Iron*: Fe is necessary for chlorophyll biosynthesis, N fixation, DNA replication and repair, and the electron transport chain (Pushnik et al., 2008; Zhang, 2014). Excess Fe inhibits root growth, impacting the uptake of other nutrients, a problem increased by anaerobic and acidic soil conditions (Becker and Asch, 2005). Under Fe deficient conditions, expression of Iron-regulated Transporter 1 (IRT1) is upregulated in roots to promote the uptake of Fe^2+^ from the rhizosphere (Vert et al., 2002). IRT1 forms a plasma membrane-localized complex with H^+^-ATPase2 (AHA2), which mediates the extrusion of protons to acidify the rhizosphere and solubilize iron, and Ferric Chelate Reductase 2 (FRO2) that reduces Fe^3+^ to Fe^2+^ for import (Connolly et al., 2003; Santi and Schmidt, 2009; Martín-Barranco et al., 2020). IRT1 transports other essential cations, including Zn, Mn, cadmium (Cd), and cobalt (Co; Vert et al., 2002). The iron transporter is ubiquitinated at the plasma membrane by the RING-type E3 IRT1 Degradation Factor 1 (IDF1), which is suggested to promote endocytosis leading to vacuolar degradation as well as turnover by the proteasome (Barberon et al., 2011; Shin et al., 2013; Dubeaux et al., 2018). Increasing concentrations of non-iron metals, such as Zn, Mn, and Co, promotes the monoubiquitination of IRT1 followed by IDF1-mediated polyubiquitination using 63-lysine linkages to generate the chain (Barberon et al., 2011; Dubeaux et al., 2018). Both modifications, monoubiquitination and polyubiquitination, decrease the levels of IRTI in the plasma membrane by promoting internalization and degradation in the vacuole. The switch to IDF1-mediated ubiquitination, in response to excess non-iron metals, is triggered by CIPK23-depedent phosphorylation of IRT1 (Dubeaux et al., 2018). AHA2 and FRO2 are also ubiquitinated; however, the modification is not induced by non-iron metals and does not promote internalization and degradation but is suggested to regulate enzyme function (Martín-Barranco et al., 2020). FER-like Iron Deficiency-induced Transcription Factor (FIT) is a key regulator among a group of basic helix–loop–helix (bHLH) transcription factors that control the expression of *IRTI* and other Fe-deficiency responsive genes (Cohen et al., 2004; Mai et al., 2016). Fe deficiency triggers a cascade of bHLH regulators that culminates in the expression and activation of FIT, which then promotes the transcription of genes involved in the mobilization and uptake of iron. Two bHLH transcription factors, bHLH105 and bHLH115, are ubiquitinated and targeted for proteasomal degradation by the RING-type E3 BRUTUS (BTS), which prohibits activation of the Fe-deficiency response in the absence of stress (Selote et al., 2015). Iron Mans (IMAs) are a family of peptides that bind to BTS under Fe-deficiency and disrupt the interaction with bHLH105 and bHLH115, allowing the transcription factors to accumulate and promote the stress response (Grillet et al., 2018; Li et al., 2021). BTS belongs to a family of hemerythrin-containing RING-type E3s, which include BRUTUS-LIKE1 (BTSL1) and BTSL2 and are similar to *O. sativa* Hemerythrin motif-containing RING- and Zinc-finger protein 1 (OsHRZ1) and OsHRZ2 (Kobayashi et al., 2013; Rodríguez-Celma et al., 2019). BTSL1 and BTSL2 regulate FIT abundance *via* proteasome-dependent degradation (Rodríguez-Celma et al., 2019). BTS, OsHRZ1, and OsHRZ2 have been shown to bind Fe and Zn, suggesting that the E3s function as metal sensors as well as negative regulators of the Fe-deficiency response (Kobayashi et al., 2013; Selote et al., 2015; Rodríguez-Celma et al., 2019). OsHRZ1 and OsHRZ2 are also required for limiting metal uptake under excess iron stress, further suggesting a role for the E3s in iron sensing (Aung et al., 2018). The RING-type E3s Ring Domain Ligase1 (RGLG1) and RGLG2 are also involved in regulating responses to iron limiting condition; however, substrates are not known (Pan et al., 2015).

*Copper*: Regulation of Cu uptake is critical because it is a key component in numerous enzymatic activities and photosynthetic processes, and high concentrations are toxic to cells (Kumar et al., 2021). In Arabidopsis, Cu uptake is achieved by a family of five high-affinity transporters, Copper Transporter (COPT)1, COPT2, COPT3, COPT5, and COPT6 (Sancenón et al., 2003 and 2004; Jung et al., 2012). Although COPT1, COPT2, and COPT6 are plasma membrane localized they interact with two closely related ER-localized proteins, Ubiquitin-associated Domain-containing Protein 2a (UBAC2a) and UBAC2b (Li et al., 2021). *UBAC2* mutants accumulate less COPT1, COP2, and COPT6 proteins, display reduced Cu root content, and increased sensitivity to Cu deficiency stress. COPT1 protein levels in *ubac2a/b* increase following inhibition of proteasome activity, suggesting regulation by the UPS. UBAC2a/b is suggested to interact with the newly synthesized COPT1 to prohibit degradation of the Cu transporter. The E3 that regulates COPT1 protein abundance is unknown.

*Boron*: B is required for synthesizing and maintaining structural integrity of cell walls and pollen viability (Camacho-Cristóbal et al., 2008). Insufficient or excess levels of boron are detrimental to crop yield and quality. Under B sufficient levels, passive transport through the cell membrane is the dominant uptake mechanism (Cervilla et al., 2009). In Arabidopsis, the uptake of B as boric acid under nutrient limiting conditions is dependent on boric acid channel protein Nodulin 26-like Intrinsic Protein5;1 (NIP5;1; Takano et al., 2006). Under B-deficiency, the boron transporter BOR1 is involved in xylem loading and translocation of B from root-to-shoot in Arabidopsis and rice (Takano et al., 2002; Nakagawa et al., 2007). Boron-induced ubiquitination of BOR1 does not target the transporter to the proteasome but promote endocytosis and vacuolar degradation (Kasai et al., 2011). High boron levels are suggested to induce a conformational change in BOR1 that triggers the attachment of a 63-lysine linked polyubiquitin chain at a previously inaccessible lysine on the C-terminal tail of the transporter (Yoshinari et al., 2021). The E3 responsible for BOR1 polyubiquitination is not yet identified.

## Conclusion

Knowledge of ubiquitin-mediated processes involved in maintaining the homeostasis of nutrients such as iron and nitrogen is well established. The UPS regulates the presence of transporters at the plasma membrane that uptake nutrients from the rhizosphere, the abundance of transporters that translocate nutrients from the root to above ground organs. The UPS also modulates the levels of regulators that control transcription of genes involved in nutrient homeostasis. However, our understanding of how the UPS functions to regulate the uptake of most nutrients, such as boron, zinc, and copper, is very limited. As research progresses the extent of ubiquitin-mediated regulation in sensing, uptake and translocations of nutrients will undoubtedly become more apparent. This may also lead to a better understanding of the mechanisms that regulate ubiquitin ligase activity and substrate engagement under different levels of nutrient availability. Considering climate change, more detailed knowledge of the role of ubiquitination in nutrient acquisition may assist with understanding how optimal nutrient status can be maintained to ameliorate the negative impact of abiotic and biotic stresses on growth and yeild.

## Author Contributions

EM and SS conceptualize the topic. EM wrote the draft and made the figures. SS edited the draft and figures. All authors contributed to the article and approved the submitted version.

## Funding

This work is supported by a Discovery grant from the Natural Sciences and Engineering Research Council of Canada (NSERC) to SLS. EM is supported by a Nova Scotia Graduate Scholarship (NSGS) and a graduate scholarship from Dalhousie University.

## Conflict of Interest

The authors declare that the research was conducted in the absence of any commercial or financial relationships that could be construed as a potential conflict of interest.

## Publisher’s Note

All claims expressed in this article are solely those of the authors and do not necessarily represent those of their affiliated organizations, or those of the publisher, the editors and the reviewers. Any product that may be evaluated in this article, or claim that may be made by its manufacturer, is not guaranteed or endorsed by the publisher.
